# Molecular and genetic characterization of the *Ry*_*adg*_ locus on chromosome XI from Andigena potatoes conferring extreme resistance to potato virus Y

**DOI:** 10.1007/s00122-018-3123-5

**Published:** 2018-05-31

**Authors:** María del Rosario Herrera, Laura Jara Vidalon, Juan D. Montenegro, Cinzia Riccio, Frank Guzman, Ida Bartolini, Marc Ghislain

**Affiliations:** 1 Applied Biotechnology Laboratory, International Potato Center, P.O. Box 1558, Lima 12, Peru; 2 International Potato Center, P.O. Box 25171, Nairobi, 00603 Kenya; 3 Present Address: Australian Genome Research Facility, University of Queensland, Brisbane, QLD 4072 Australia; 4 Present Address: Postgraduate Program in Cellular and Molecular Biology (PPGBCM) - Biotechnology Center (CBiot), UFRGS, Bento Gonçalves Ave. 9500/Building, 43431 Porto Alegre, RS Brazil; 5 Present Address: Laboratorio de Biología Molecular del Servicio Nacional de Sanidad Agraria (SENASA), Av La Universidad 1915, La Molina, Lima 12, Peru

## Abstract

**Key message:**

We have elucidated the Andigena origin of the potato *Ry*_*adg*_ gene on chromosome XI of CIP breeding lines and developed two marker assays to facilitate its introgression in potato by marker-assisted selection.

**Abstract:**

Potato virus Y (PVY) is causing yield and quality losses forcing farmers to renew periodically their seeds from clean stocks. Two loci for extreme resistance to PVY, one on chromosome XI and the other on XII, have been identified and used in breeding. The latter corresponds to a well-known source of resistance (*Solanum stoloniferum*), whereas the one on chromosome XI was reported from *S. stoloniferum* and *S. tuberosum* group Andigena as well. To elucidate its taxonomic origin in our breeding lines, we analyzed the nucleotide sequences of tightly linked markers (M45, M6) and screened 251 landraces of *S. tuberosum* group Andigena for the presence of this gene. Our results indicate that the PVY resistance allele on chromosome XI in our breeding lines originated from *S. tuberosum* group Andigena. We have developed two marker assays to accelerate the introgression of *Ry*_*adg*_ gene into breeding lines by marker-assisted selection (MAS). First, we have multiplexed RYSC3, M6 and M45 DNA markers flanking the *Ry*_*adg*_ gene and validated it on potato varieties with known presence/absence of the *Ry*_*adg*_ gene and a progeny of 6,521 individuals. Secondly, we developed an allele-dosage assay particularly useful to identify multiplex *Ry*_*adg*_ progenitors. The assay based on high-resolution melting analysis at the M6 marker confirmed *Ry*_*adg*_ plex level as nulliplex, simplex and duplex progenitors and few triplex progenies. These marker assays have been validated and can be used to facilitate MAS in potato breeding.

## Introduction

Potato viruses are increasingly a source of concern with less predictable insect vector population due to climate changes not only directly on reducing crop yield, but also because they spread through healthy-looking tubers (Solomon-Blackburn and Barker 2001). Around 40 viruses infect cultivated potatoes in the field with the most ubiquitous being potato virus Y (PVY), potato leafroll virus (PLRV) and potato virus A (PVA) which are found worldwide (Valkonen 2007). The most sustainable way to reduce losses caused by potato viruses is using resistant cultivars (Swiezynski 1994). Virus resistance genes in potato are of various types, immunity, extreme resistance and hypersensitivity (Valkonen 1994). PVY can reduce potato yield up to 80% (de Bokx and Huttinga 1981; Hane and Hamm 1999; Rykbost et al. 1999; Nolte et al. 2004). Extreme resistance to PVY has been found in *Solanum tuberosum* L. group Andigena (*Ry*_*adg*_) (Muñoz et al. 1975; Gálvez and Brown 1980; Gálvez et al. 1992), *S. stoloniferum* (*Ry*_*sto*_), *S. chacoense* (*Ry*_*chc*_*)*, *S. demissum* and *S. hougassi* (Cockerham 1970). Genes for resistance to PVY have been mapped from *S. chacoense, S. stoloniferum, S. tuberosum* group Andigena and *S. tuberosum* group Tuberosum (Simko et al. 2007). The *Ry*_*adg*_ gene has been localized on chromosome XI by Hämäläinen et al. (1997). Brigneti et al. (1997) located the PVY resistance gene on chromosome XI using a large population derived from the cross 83W28-50 × I-1039 (I-1039 carries the *Ry*_*sto*_ gene) and referred to it as *Ry*_*sto*_. However, Gebhardt and Valkonen (2001) expressed doubts about the origin of the material used in this study. The *Ry*_*sto*_ gene (Song et al. 2005) as well as the gene named *Ry*-*f*_*sto*_ (Flis et al. 2005) was mapped on chromosome XII. It is likely that both genes are allelic forms of the same gene because of similar location on the potato genetic map, but their DNA sequences are still lacking and need to be matched. The *Ry*_*chc*_ gene was identified on chromosome IX (Hosaka et al. 2001; Sato et al. 2006). Also, hypersensitivity genes have been mapped on the potato map: *Ny*_*tbr*_ gene on chromosome IV (Celebi-Toprak et al. 2002), *Ny*-*1* on chromosome IX (Szajko et al. 2008) and *Ny*-*2* on chromosome XI (Szajko et al. 2014). *Ny*-*Smira* gene from cultivar Sarpo Mira was mapped to a location on the chromosome IX corresponding to *Ry*_*chc*_ and *Ny*-*1* genes for PVY resistance. (Tomczyńska et al. 2014). Recently, *Ny*_*(o,n)sto*_ gene from *S. stoloniferum* was identified in potato cultivars in a position corresponding to the *Ry*_*sto*_ on chromosome XI described by Brigneti et al. (1997) (Van Eck et al. 2017).

Several molecular markers have been developed for detection of these genes. Hereafter, we will focus on the *Ry*_*adg*_ gene which is used in our potato breeding program for adaptation to lowland tropics (Mendoza et al. 1996). Four RFLP markers were identified closely linked to the *Ry*_*adg*_ locus and tested in tetraploid potatoes (Hämäläinen et al. 1997). A year later, the same group identified two PCR-amplified genomic fragments, ADG1 and ADG2, closely linked to *Ry*_*adg*_ (Hämäläinen et al. 1998). Polymorphism between ADG2 sequences from PVY-resistant and -susceptible parental lines was used to develop a sequence-characterized amplified region (SCAR) marker RYSC3 (Kasai et al. 2001). Additional linked AFLP markers (M5, M6, M17, M33, M35 and M45) and one RFLP marker (GP259) were identified in a different segregating population (Brigneti et al. 1997).

Marker-assisted selection (MAS) offers important advantages over conventional breeding methods: time saving by reducing the number of breeding steps, selection at an early generation stage, reduced number of lines to be tested, selection of individual plants based on their genotype and gene ‘pyramiding’ (Collard et al. 2005; Collard and Mackill 2008; Slater et al. 2013).

Most DNA marker systems are developed for diploid material and are not easily applicable for tetraploid potato material (Cernák et al. 2008; De Koeyer et al. 2010). To develop molecular tools to be used for MAS for PVY resistance, the applicability of some markers linked to *Ry*_*adg*_ genes has been evaluated in different genetic backgrounds. The ADG2 marker was used as an RFLP probe and tested on potato cultivars and breeding lines and results indicated this marker as useful tool for MAS (Shiranita et al. 1999). Whitworth et al. (2009) evaluated the effectiveness of RYSC3, RYSC4 and ADG2 markers to identify PVY resistance in the USDA-ARS Aberdeen Idaho Potato Breeding Program. Dalla Rizza et al. (2006) reported the application of M45 and RYSC3 markers in MAS for PVY resistance in the National Breeding Program of Uruguay. To implement MAS for PVY resistance in the Oregon Potato Breeding Program, the RYSC3 marker and the CAPS marker ADG2 were used and confirmed as tools to identify PVY-resistant clones (Ottoman et al. 2009). Appacale (Spain), a public breeding company, used RYSC3 and STM0003 markers to select breeding clones resistant to PVY (Ortega and Lopez-Vizcon 2012). The SCAR markers RYSC3 and YES3-3 were used for application of MAS in a conventional potato breeding program in the University of Wisconsin, USA (Fulladolsa et al. 2015).

Typically, a conventional potato breeding program starts with a large breeding population which is then subject to phenotypic recurrent selection over a number of generations, taking 10–12 years to produce one new variety (Bradshaw and Mackay 1994; Lindhout et al. 2011; Gebhardt 2013; Ortega and Lopez-Vizcon 2012). Extreme resistance to economically important potato diseases is often controlled by major genes which are mostly simplex, thereby transmitting the resistance only to half of their progeny. Thus, the production of multiplex parents is an advantage for increasing the frequency of favorable alleles (Bradshaw and Mackay 1994). The allele dosage of valuable progenitors in potato breeding is determined through the time-consuming progeny test and analyzing the resulting segregation ratio (Cockerham 1970; Bradshaw and Mackay 1994; Mendoza et al. 1996; Slater et al. 2014). Hence, a marker system that allows to distinguish several allelic combinations and their dosage would be advantageous for tetraploid potato breeding (De Koeyer et al. 2010).

High-resolution melting (HRM) is a simple post-PCR technique for determining sequence variations within PCR amplicons based on their dissociation profile. HRM is applied to genotype known sequence variants by using unlabeled probes and asymmetric PCR producing melting curves for each genotype. In potato, HRM was proven to be very useful for identification of tetraploid clones with desirable alleles (De Koeyer et al. 2010).

In the present study, we validated markers linked to the *Ry*_*adg*_ gene on chromosome XI to diagnose for extreme resistance to PVY of potato clones deriving from breeding programs which use *S. tuberosum* group Andigena germplasm as source of resistance to PVY. We further characterized the *Ry*_*adg*_ gene locus with respect to the similarity and putative origin of the resistant alleles to PVY. We focused on the *Ry*_*adg*_ loci because it is currently being used in CIP breeding program and is effective against all known strains of PVY including PVY^NTN^. This paper describes the development of a reliable, cost-effective and a mid-throughput marker assay of the RYSC3, M45 and M6 markers to be used in large-scale progeny screening for PVY resistance, and a molecular assay of the M6 marker for assessing the dosage of resistant alleles of *Ry*_*adg*_ also referred to as a “plex” assay. Both are suitable for MAS in potato breeding programs to facilitate the introgression of the *Ry*_*adg*_ gene into promising potato clones.

## Materials and methods

### Plant materials

Biological materials with distinct and relevant genotypes and phenotypes have been selected from CIP germplasm, and breeding stocks referred to as the validation panel hereafter: ten progenitors and varieties with extreme resistance to PVX and PVY (DXY.7, DXY.10, DXY.15, TXY.2, TXY.11, Costanera, UNICA with the *Ry*_*adg*_ gene, I-1039 with the *Ry*_*sto*_ gene; Pirola, Bzura with the *Ry*-*f*_*sto*_ gene), four PVY-susceptible varieties (Alkanche, Atlantic, Bintje, and Perricholi) and three potato clones and varieties with hypersensitive resistance to PVY (A6, Desiree, Granola with *Ny* gene). In addition, we used two accessions of *S. stoloniferum*, one resistant OCH14135 with an unmapped *Ry*_*sto*_ gene and one susceptible TARN187. The 251 accessions of the *S. tuberosum* group Andigena were obtained from CIP genebank and chosen randomly.

We developed a segregating population, referred to as the CL population, for the *Ry*_*adg*_ locus comprising 6521 individuals from a cross between the potato variety Costanera (CIP379706.27) with extreme resistance to PVY mediated by the *Ry*_*adg*_ gene and the susceptible LBr-43 clone (CIP387170.9). All plants were grown in a controlled environment at 20 °C, 12 h light and between 70 and 100% relative humidity. Seedling trays with 96 wells were used to establish a one-to-one correspondence between plants and DNA extracted and analyzed in 96-well plates for marker analyses by PCR.

For the allele-dosage assay, we used *Ry*_*adg*_ bearing varieties and clones genotyped by genetic analysis of their test-cross progenies as RRRr (TXY.2 and TXY.11); RRrr (DXY.7, DXY.10 and DXY.15); Rrrr (Costanera and UNICA) (Mendoza et al. 1996; Mihovilovich et al. 1998). Two F1 hybrid populations were developed: (1) CT population of 220 genotypes from a cross between the variety Costanera (C) and the breeding clone TXY.2 (T); and (2) LT population of 163 genotypes from a cross between TXY.2 (T) and LBr-43 (L).

### PVY resistance assay

The virus infection was conducted using six plants of each potato genotype inoculated with strain “O” of PVY with three of them mechanically and the other three by grafting. Thirty days after inoculation, symptoms were observed and ELISA tests were carried out by the CIP virology unit (Clark and Adams 1977).

### DNA extraction

Total DNA was obtained by using standard protocols at CIP (Herrera and Ghislain 2000). For the segregating population, DNA extraction was done using leaves from 2-week-old seedlings. Using a small paper punch, two leaf discs (0.6 mm in diameter) were cut and placed in its corresponding place into the well of a 96-well PCR. Plates were put on ice and stored at − 70 °C. DNA extraction was carried out based on the method of Dayteg et al. (1998) described by von Post et al. (2003) and adapted to the 96-well plate for medium-throughput extraction. Forty microliters of 0.25 M NaOH was added to each well plate directly on the tissue. The plate was placed in a 95 °C water bath for 1 min. Samples were crushed with a 12-channel pipette and neutralized with 120 µl of 0.1 M Tris–HCl, pH 8.0. Plates were centrifuged at 900 rpm for 5 s. The aqueous phase contained the DNA which was used directly for PCR.

### PCR assay

Single amplicon reactions were made using 100 ng of potato DNA in 20 µl reaction volume [1× PCR buffer, 2.5 mM MgCl_2_, 0.2 mM each dNTP, 0.4 µM primer F, 0.2 µM primer R], 0.5 µl Taq Polymerase (1 unit/µl), and run on a PT100 thermocycler (MJ Research, USA). CAPS markers were obtained by adding 1 µl of the corresponding restriction enzyme (10 units/µl) directly to the PCR reaction after the amplification was terminated and incubated 1 h at 37 °C. Primers corresponding to *Ry*_*adg*_ *and Ry*_*sto*_ markers, restriction enzymes for CAPS markers, SCAR marker RYSC3, M45 and M6 markers are indicated in Table 1. Reactions were loaded onto standard 1% agarose gels using Tris–borate buffer. Amplification products were visualized using ethidium bromide fluorescence under UV light.

**Table 1:** Primer sequences and restriction conditions to detect five molecular markers associated with *Ry* genes

Marker	*Ry* gene	Primer name	Sequence (5′–3′)	T°a	Digestion	Amplicon size (bp)	References
*RY*SC3	*Ry*_*adg*_	3.3.3 S	ATACACTCATCTAAATTTGATGG	60	_	321	Kasai et al. (2001)
		ADG23R	AGGATATACGGCATCATTTTTCCGA				
M5	*Ry*_*sto*_	M5-m	GCTGTTCACAATGGGAACATGG	60	*Mse*I/BSA	485	Brigneti unpublished
		M5-p	CATACAAACTACTTCTACCACG				
M6	*Ry*_*sto*_	M6 Rsa	TCCGAAATGTTTGGGCTGACATC	55	*Rsa*I	1126	Brigneti unpublished
		M6 Taq	AAGGGATCCAAAAAGGTGGTTCA				
M17	*Ry*_*sto*_	M17-m	GACTGCTTTCTCTCCACGTGGC	60	*Pst*I	250	Brigneti unpublished
		M17-l	GATCACAGATGTTTTACCTTCGATG				
M45	*Ry*_*sto*_	M45-m	CCTAGTTTCTGAGCATGTAATTTC	61	_	495	Brigneti unpublished
		M45-p	TGCAGCTATTCAAAACACATAAGG				
M6	*Ry*_*adg*_	M6F1	ACATGATATAAGTTGATATGGAGAAT	60	_	994	This article
		M6R4	GTGCTTTGTCTTTTCTGCATGTA				
M45	*Ry*_*adg*_	M45F1	TGGAGTATTTGGATCTAAGGG	61	_	268	This article
		M45R1	AACACATAAGGAGCGATG				
M6	*Ry*_*adg*_	P2F1	TAGATACGCCACTCCACATA	60.5	_	135	This article
		P2R1	GAACCCATCCGTGATAAAT				
		P2	GCTGCTCGGGGTCACCAC				

### Sequencing M45 and M6 amplicons

M45 and M6 amplicons were purified from agarose gels using the Wizard SV Gel and PCR Clean-Up System (Promega) following the manufacturer’s instructions. Purified fragments were then cloned into plasmid vector using the pGEM-TEasy Vector System (Promega) according to the manufacturer’s instructions. Plasmid DNA was then extracted using Wizard Plus SV Minipreps (Promega) according to the manufacturer’s manual. These DNA were sequenced both forward and reverse sense by Macrogen Inc. From 10 to 20 randomly chosen cloned amplicons were sequenced.

### Sequence analyses

Forward and reverse nucleotide sequences from the same amplicon cloned were aligned using the software Codon Code Aligner. Due to sequencing errors by the Taq polymerase used in the present study, we used identical sequences obtained from sequencing amplicons cloned from several PCR amplifications. Analysis of nucleotide sequence identity was performed using the software Genious R9.0.5, whereas a dendrogram based on multiple sequence alignment was developed using the unweighted pair group method with arithmetic mean (UPGMA). The 12 M6 allelic sequences used to construct the dendrogram were deposited in Genbank under accession numbers MG979714 to 25.

### High-resolution melting primers and probes

Primers and probes used the M6 sequences of the allele associated with the resistance, referred to as M6R, and of the three alleles associated with the susceptibility (M6S1, 2, 3, and 4) to PVY at the M6 locus. Primer3 software was used with default parameters for primers producing an amplicon less than 250 bp (Rozen and Skaletsky 2000). The probes have a Tm of about 5–10 °C above the Tm of the primers. Eight primer and probe sets were designed for further evaluation.

### Asymmetrical unlabeled probe genotyping PCR

PCR was performed using 60 ng of potato DNA in 10 µl reaction volume [1× PCR buffer, 2.5 mM MgCl_2_, 0.2 mM each dNTP, 0.02 µM forward primer, 0.2 µM reverse primer, 0.2 µM probe (Integrated DNA Technologies)] and 0.5 unit of Taq polymerase (Invitrogen). PCR was performed on a PTC-100 thermocycler (MJ Research Inc.) in 96-well plates. The program used was the following: 2 min at 94 °C, followed by 55 cycles of 40 s at 94 °C, 40 s at annealing temperature (Ta), and 45 s at 72 °C, then 5 min at 72 °C as a final extension step. Finally, the reaction was cooled to 25 °C. PCR products were evaluated on a 1% agarose gel electrophoresis. The asymmetric PCR was performed in Hard-Shell PCR plates, 96 wells, thin walled (Bio-Rad, cat. HSP9665) with the addition of 1× LC Green (BioFire Defense). The reaction mixture was overlaid with 15 µl of mineral oil and the plates were covered with adhesive film Microseal B (Bio-Rad, cat. MSB1001). An extra step of 30 s at 94 °C was added to the PCR program to allow the formation of the hybrid between the probe and the strand in excess.

### HRM of unlabeled probes analysis

After PCR, the plates were taken to a high-resolution LightScanner (Idaho Technology, Salt Lake City, UT), an instrument dedicated to melting curve analysis. DNA was melted from 45 to 95 °C with a hold temperature of 42 °C. Acquired data were analyzed with the Unlabelled Probes module of the LigthScanner software. As a first step, blanks and samples that did not amplify were excluded from the analysis. Melting curves were normalized at 57.6–75.8 for the validation panel and 60.8–75.8 °C for the segregating populations. Melting curves were displayed as derivative melting peaks. Then, samples with similar melting profile were grouped using sensitivity at normal + 0.9. Finally, a Tm for each peak was calculated.

## Results

### Molecular markers to assist and accelerate selection of *Ry*_*adg*_ gene for extreme resistance to PVY

Phenotypic data of the 17 potato varieties were reconfirmed using symptoms and DAS-ELISA. Symptoms were mosaic followed by necrosis in susceptible varieties and chlorotic spots or rings in hypersensitive varieties, whereas no symptoms were observed for plants with extreme resistance to PVY. Only the varieties Atlantic and Alkanche displayed chlorotic rings and spots, respectively, instead of necrosis. Detection of PVY by serology, DAS-ELISA, was negative for all symptomless varieties and virus-resistant progenitors (UNICA, Costanera, DXY.10, DXY.7, DXY.15, TXY.2, TXY.11, I-1039, Pirola, Bzura) and positive for all others except the varieties Perricholi and Desiree (Table 2). In the case of the *S. stoloniferum* accessions, both symptom and serology analyses confirmed that OCH14135 and TARN187 are extremely resistant and susceptible to PVY, respectively.

**Table 2:** Resistance to PVY and association of *Ry* markers of 17 selected potato varieties, breeding clones and two accessions of the wild species *Solanum stoloniferum*

CIP number	Cultivar	Gene	Symptoms		DAS-ELISA		Molecular Markers				
			Mech.*	Graft	Mech.*	Graft	M17	M45	M5	M6	RYSC3
800,965	A6	*Ny*	Necrosis	Necrosis	1	1	–	–	–	–	+
800,048	Desiree	*Ny*	Necrosis	Necrosis	0	1	–	–	–	–	–
800,959	Granola	*Ny*	Necrosis	Necrosis	1	1	–	–	–	–	–
704,790	Alkanche	–	Necrosis	Chlorotic spots	1	1	–	–	–	–	–
800,827	Atlantic	–	Necrosis	Chlorotic rings	1	1	–	–	–	–	–
801,069	Bintje	–	Necrosis	Necrosis	1	1	–	–	–	–	–
374,080.5	Perricholi	–	Necrosis	Necrosis	0	0	–	–	–	–	–
391,894.7	DXY.7	*Ry*_*adg*_	–	–	0	0	+	+	+	+	+
391,895.1	DXY.10	*Ry*_*adg*_	–	–	0	0	+	+	+	+	+
391,896.15	DXY.15	*Ry*_*adg*_	–	–	0	0	+	+	+	+	+
393,613.2	TXY.2	*Ry*_*adg*_	–	–	0	0	+	+	+	+	+
393,617.1	TXY.11	*Ry*_*adg*_	–	–	0	0	+	+	+	+	+
379,706.27	Costanera	*Ry*_*adg*_	–	–	0	0	+	+	+	+	+
392,797.22	UNICA	*Ry*_*adg*_	–	–	0	0	+	+	+	+	+
676,008	I-1039	*Ry*_*sto*_	–	–	0	0	+	+	+	+	–
800,953	Bzura	*Ry*-*f*_*sto*_	–	–	0	0	–	–	–	–	–
800,957	Pirola	*Ry*-*f*_*sto*_	–	–	0	0	–	–	–	–	–
760,738	TARN187	–	Mosaic	Mosaic	1	1	+	+	–	–	+
761,884	OCH14135	*Ry*_sto*_	–	–	0	0	+	–	–	+	–

*Mechanical infection

All potato materials were analyzed with the five *Ry*_*adg*_ markers: the two SCAR markers RYSC3 and M45, and the three CAPS markers M5, M6 and M17 (Table 2). These markers were chosen because of their proximity to the *Ry*_*adg*_ gene. According to Brigneti et al. (1997), genetic distance between M17 and M45/M5 is 0.3 cM and between M45/M5 and M6 also 0.3 cM. Jara Vidalon (2010) estimated at 0.2 cM the genetic distance between RYSC3 and M45/M5 and between M45/M5 and M6 to be 0.05 cM, using a much larger segregating population of 6521 individuals. Only in the 2010 genetic mapping study, one recombinant was found between the PVY extreme resistance gene and the M45/M5 makers. Hence, these are closest markers to the *Ry*_*adg*_ gene which is located between M6 and M45.

The two varieties with extreme resistance and five virus resistant progenitors that carry *Ry*_*adg*_ (UNICA, Costanera, DXY.10, DXY.7, DXY.15, TXY.2 and TXY.11) presented the marker allele associated with resistance for all five markers. In the case of the three varieties with extreme resistance that carry *Ry*_*sto*_ gene from *S. stoloniferum*, I-1039 presented marker alleles associated with resistance for M5, M6, M17 and M45 but not for RYSC3, whereas Pirola and Bzura did not amplify any of these markers. The latter result was expected because virus resistance in both varieties is determined by the *Ry*-*f*_*sto*_ gene (Flis et al. 2005; Song et al. 2005). Of the hypersensitive resistant and susceptible potato materials, none presented a marker allele associated with resistance except the clone A6 for only the RYSC3 marker (used for PVY strain indexing). The two accessions of *S. stoloniferum* produced amplicons corresponding to marker alleles associated with resistance except for M5. Both genotypes displayed the same resistant allele for M17, whereas for RYSC3 and M45 the marker allele associated with resistance was detected in the susceptible accession TARN187. The resistant accession OCH14135 presented the marker associated with resistance for M6. However, the PVY resistance gene(s) in OCH14135 cannot be clearly related to the one on chromosome XI or XII unless a segregation analysis is done.

### Comparison of *Ry*_*adg*_ gene locus on chromosome XI in germplasm resistant to PVY

M6 and M45 amplicons from PVY-resistant varieties UNICA and Costanera (bearing *Ry*_*adg*_), I-1039 (bearing *Ry*_*sto*_), and the accession OCH14135 of *S. stoloniferum* were sequenced. This *sto* accession is resistant to PVY supposedly due to the *Ry*-*f*_*sto*_ gene located on chromosome XII. Marker analyses did not suggest there was another *Ry*_*sto*_ gene on chromosome XI because M45, closest to *Ry*_*adg*_ (Brigneti et al. 1997; Jara Vidalon 2010), did not amplify, and only M6 primers produced an amplicon of the expected size (Table 2).

**Fig. 1:**
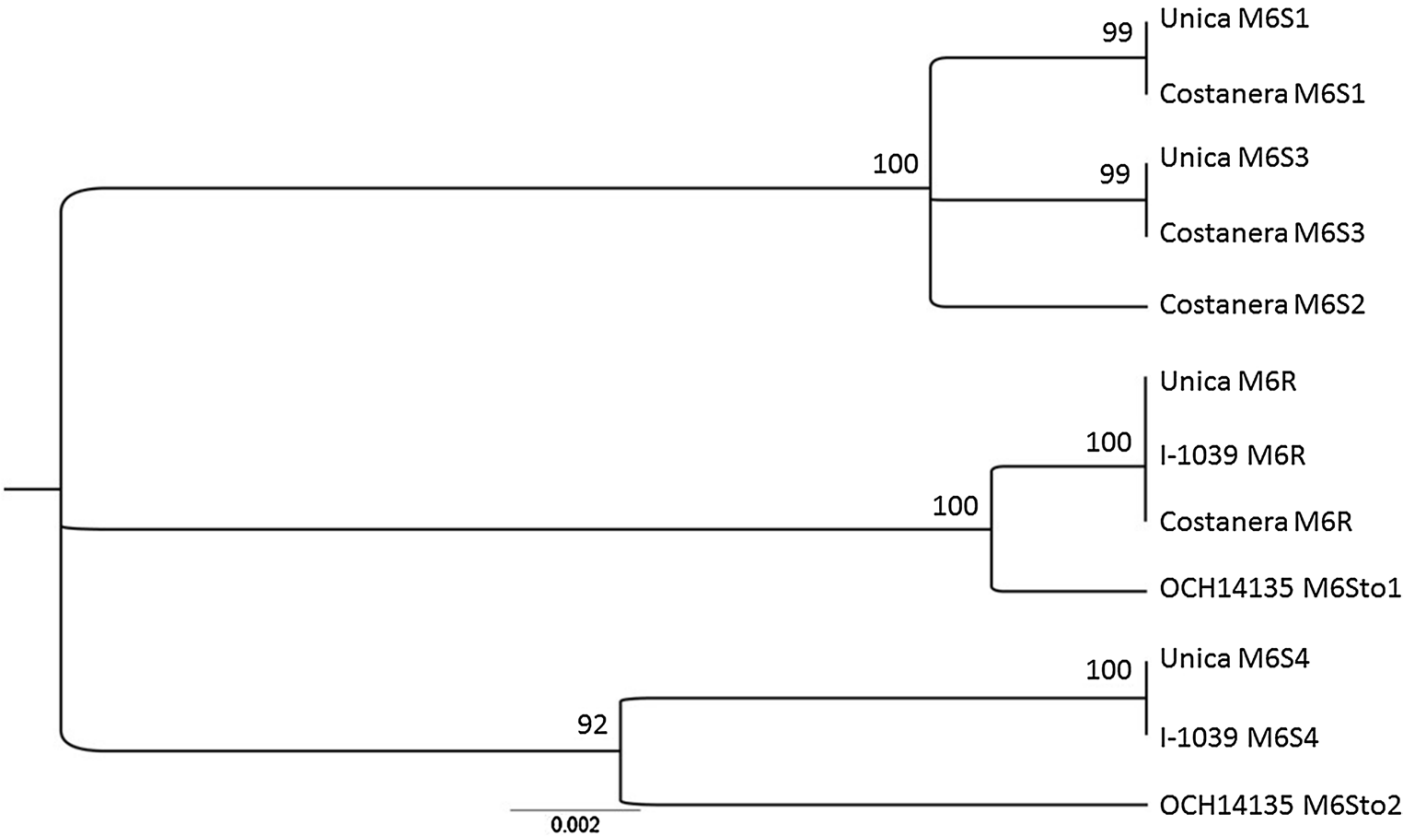
Dendrogram of nucleotide sequences of the M6 locus from Costanera, UNICA, I-1039 and the *S. stoloniferum accession* OCH14135. Numbers represent branching frequency with bootstrapping set at 100

The M6 marker provided amplicons of the expected size of 1126 bp for all genotypes tested. Sequence analyses from cloned amplicons confirmed that they represented more than one allele. A total of seven M6 alleles were identified: one associated with the resistant *Ry*_*adg*_ allele(s), referred to as M6R (identified by the presence of the *Rsa*I site); four with the susceptible allele(s) referred to as M6S1, 2, 3, 4; and two from *S. stoloniferum* referred to as M6sto1, 2. As expected, each tetraploid potato presented a maximum of four alleles. The M6R allele was identical between Costanera, UNICA, and I-1039, whereas it presented seven SNPs and two deletions (99% sequence identity) with M6sto1 of the OCH14135 accession of *S. stoloniferum* (Fig. 1).

The M45 sequences of 495 bp were identical for each genotype and almost identical between Costanera, UNICA and I-1039 (only one SNP), suggesting that these varieties shared the same M45 allele associated tightly with the PVY resistance gene.

Potato landraces of the *S. tuberosum* group Andigena were screened with the RYSC3, M6 and M45 markers to search for native (unbred) potatoes bearing the *Ry*_*adg*_ gene. Out of 251 landraces, we found one landrace, Pisha Milpo, positive for all three alleles. Chloroplastic and nuclear SSR marker fingerprinting was done to verify the taxonomic assignment of this genotype to the Andigena group and its extreme resistance to PVY was confirmed as well (data not shown).

### Validated multiplex PCR assay of *Ry*_*adg*_ markers

**Fig. 2:**
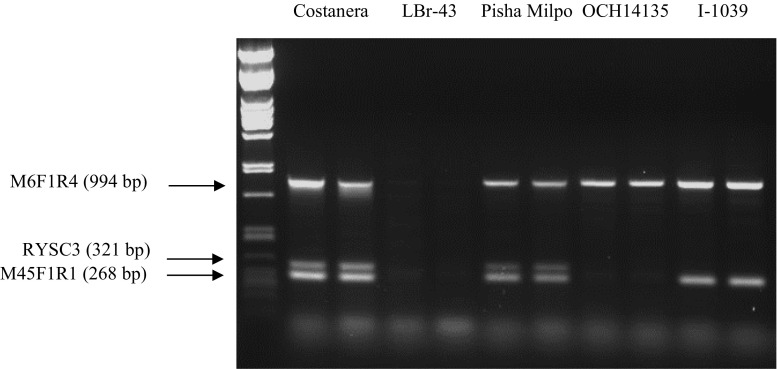
Multiplex PCR for RYSC3, M6 and M45 markers using Costanera (extreme resistance, *Ry*_*adg*_ from Andigena group), LBr-43 (susceptible to PVY), Pisha Milpo (extreme resistance, landrace of Andigena group), OCH14135 (extreme resistance, *Solanum stoloniferum*) and I-1039 (extreme resistance, *Ry*_*sto*_ from *S. stoloniferum*) (2 repetitions-lanes per genotype)

In accordance with Henegariu et al. (1997), we tested various parameters to optimize multiplex PCR. Potato clones and varieties (Costanera, LBr-43, Pisha Milpo, I-1039 and OCH14135) previously genotyped for these markers were used as validation panel. We evaluated concentrations of PCR buffer, MgCl_2_, primers, Taq polymerase and amount of DNA. PCR cycling conditions were also assessed. We redesigned the M6 and M45 primers based on sequence analysis of several potato cultivars and identified primer M6F1-M6R4 and M45F1-M45R1 combinations to be the best ones (Table 3). Adaptation to the 96-well plate format of the Dayteg protocol for DNA extraction allowed us to process 384 samples within 1.5 h. In agreement with Zhang et al. (2003), the PCR conditions for each marker locus were used as base for the subsequent optimization of the multiplex PCR. The RYSC3, M45 and M6 primers were added in equimolar amounts. Results showed that the amplification for the three markers were not uniform and suggested that primer concentration needed to be adjusted. Concentration of primers showing weak amplification was increased, while concentration of primers with stronger amplification was decreased. Best primer concentrations were 0.6 μM for M6 marker, 0.15 μM for RYSC3 marker and 0.5 μM for M45 marker. The optimal amount of DNA was determined to be 2 µl of undiluted DNA (between 10 and 20 ng). The annealing temperature was tested at 55 and 60 °C with best results obtained at 55 °C. Finally, the extension temperature was optimized as 65 °C for 4 min. Optimized amplification conditions for multiplex PCR were confirmed using the validation panel (Table 3; Fig. 2). The same multiplex PCR and optimized amplification conditions were successfully on the CL population to quickly identify recombinants in the M45–RYSC3 interval where the *Ry*_*adg*_ gene lies (data not shown, Jara Vidalon 2010).

**Table 3:** Optimized amplification conditions for multiplex PCR for RYSC3, M6 and M45 makers

	Final concentration	PCR conditions
NFW		Start: 94 °C × 1 min
M6F1	0.6 µM	30 cycles of 3 steps: 94 °C × 30 s
M6R4	0.6 µM	60 °C × 1 min
ADG23R	0.1 µM	65 °C × 4 min
3.3.3 s	0.1 µM	Last:
M45F1	0.3 µM	65 × 60 s
M45R1	0.3 µM	
10X PCR buffer (25 mM MgCl_2_)	1×	
dNTPs (5 mM)	0.2 mM	
Taq polymerase	0.5 U	
Genomic DNA	10–100 ng

### Validation of the HRM allele-dosage assay and its use in two *Ry*_*adg*_ resistance-derived progenies

**Fig. 3:**
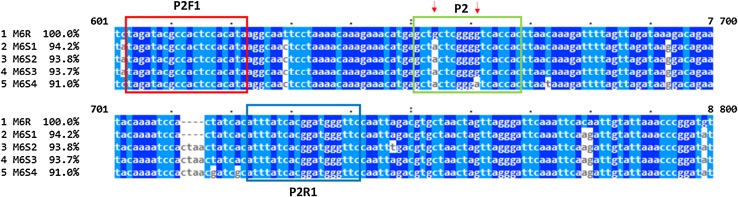
Primer probes M6P2 of the HRM allele-dosage assay. Alignment of DNA sequences of the resistant and susceptible alleles of M6 marker. Annealing sites of primers (P2F1 and P2R1) and probe (P2) are boxed. The arrows point at positions where the sequence varies between the five sequences

**Fig. 4:**
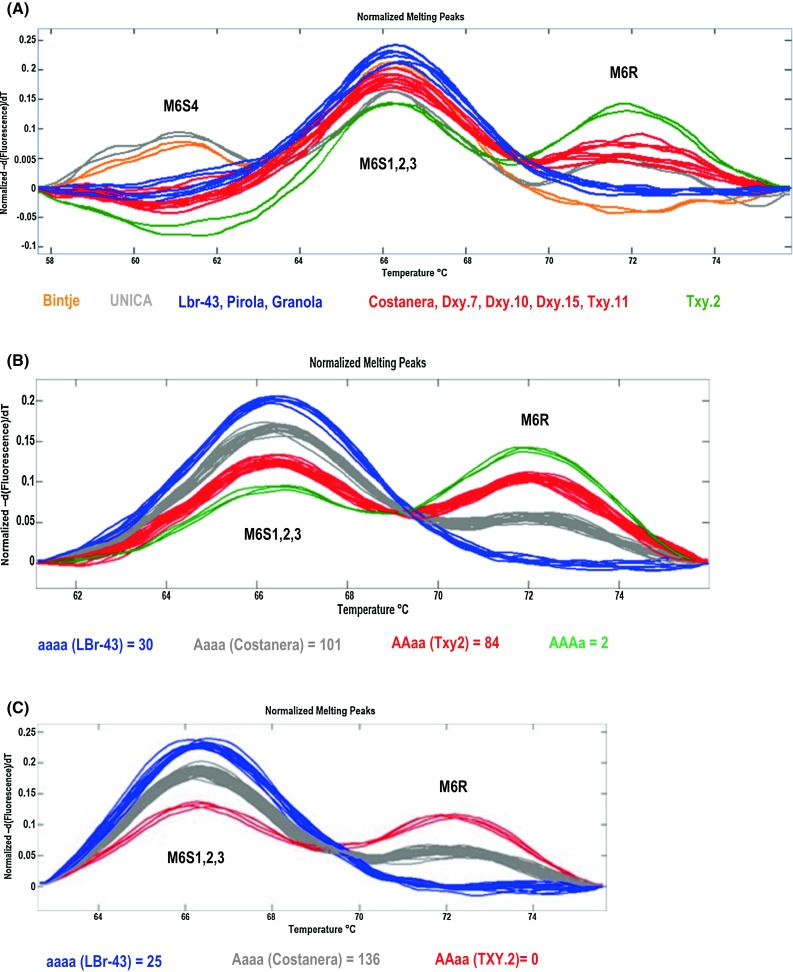
High-resolution melting allele-dosage assay at the M6 locus for: **a** the potato validation panel; **b** the progeny of Costanera with TXY.2 (CT) including LBr-43 clone as nulliplex control; and **c** the progeny of TXY.2 with LBr-43 clone (TL). The number of genotypes for each group is included

Two PVY-susceptible varieties (LBr-43 and Bintje), the hypersensitive resistant variety Granola and the resistant variety Pirola carrying the *Ry*-*f*_*sto*_ gene were used as negative controls (negative for RYSC3, M6 and M45). Using the M6 allelic sequences, we tested eight different sets of primers and probes on the validation panel. Only one probe, referred to as M6P2, could be amplified in the asymmetric PCR for all tested potato materials and was selected for further HRM analysis. This primer–probe set amplifies a 135 bp fragment with a Tm of ~ 80 °C. The probe is 18 bases long and detects two SNPs, a G in position 3 and a G in position 10, which are specific for the resistant allele (Fig. 3). The HRM allele-dosage assay on the validation panel revealed three probe melting peaks that represent different sequences in the probe region (Fig. 4a). Since the probe was designed to perfectly match the resistant allele (M6R), the peak at higher melting temperatures (72 °C) within the probe melting region indicates the presence of the target allele. The melting peak at 66.5 °C represents the susceptible alleles M6S1, M6S2 and M6S3, which have the same probe sequence and differs in one nucleotide from the resistant allele. The third melting peak at 61 °C corresponds to the susceptible allele M6S4 that differs in two nucleotides from the probe sequence of the resistant allele. Peak heights have been used to estimate dosage within the region of the probe.

Five profiles could be distinguished using the potato validation panel (Fig. 4a): First, the susceptible clone LBr-43, the resistant clone carrying *Ry*_-_*f*_*sto*_ (Pirola) and the hypersensitive resistant clone Granola had the same profile with only one peak that corresponds to the three susceptible alleles M6S1,2,3. Second, the Costanera clone (simplex) had the same profile as all duplex clones and the triplex clone TXY.11 with a high peak corresponding to the three susceptible alleles M6S1,2,3 and a small peak assigned to the resistant allele (M6R). Third, the *Ry*_*adg*_ triplex clone TXY.2 presented two peaks (M6R and M6S1,2,3) with equal height, which suggests that this is apparently a duplex. Fourth, the simplex clone UNICA showed three peaks, with the M6S4 and M6R alleles having the lowest peaks and the M6S1,2,3 alleles the highest peak. Finally, fifth, the susceptible clone Bintje displayed a high peak for the three susceptible M6S1,2,3 alleles and a low peak for the M6S4 allele. The results of the HRM-allele dosage at the M6 locus did not coincide fully with the duplex and triplex progenitors of the validation panel deduced from progeny segregation. To elucidate the reasons behind this discrepancy, the simplex by duplex (or triplex) CT population was developed.

### The HRM allele-dosage assay on the CT population

The allele-dosage-sensitive assay on the CT population, which included the susceptible clone LBr-43 as nulliplex control, displayed two clearly separated peaks corresponding to the resistant and the three susceptible M6S1,2,3 alleles (Fig. 4b). Four well-defined groups were identified corresponding to nulliplex, simplex, duplex and triplex. Of the 220 individuals, three genotypes were not considered because of inconsistent results or bad amplification. Thirty genotypes were nulliplex, 101 simplex, 84 duplex and 2 triplex. Again, in this CT population TXY.2 appeared to be a duplex for the resistant allele of *Ry*_*adg*_ gene. The abundance of nulliplex and the absence of quadruplex, expected in a simplex by a duplex cross but not in a simplex by triplex, confirmed TXY.2 as a duplex progenitor. However, the allelic distribution in this population is different from the one theoretically expected in a cross between a simplex and a duplex (1N:5S:5D:1T). The Chi square goodness of fit test (*x*^2^ = 23.854; *p *< 0.001; *p* ≤ 0.05) rejected the hypothesis that the observed values correspond to a cross between a simplex and a duplex. A second hypothesis which could explain the observed results has been formulated: unviable gametes when homozygous for the *Ry*_*adg*_ resistant gene result in a segregation (1N:5S:4D). The Chi square goodness of fit test (*x*^2^ = 3.8; *P *= 0.15; *p* ≤ 0.05) failed to reject this hypothesis (Table 4a). However, the identification of two rare triplex of the *Ry*_*adg*_ resistant allele suggests limited rather than complete loss of viability of gametes homozygous for the resistant allele of the *Ry*_*adg*_ gene.

**Table 4:** Viability of homozygous alleles for *Ry*_*adg*_ tested by Chi squared tests on distribution of allele dosage in two populations

A^1^			
Female gametes	Male gametes		
	*1/6 RR*	4/6 Rr	1/6 rr
3/6 Rr	*3/36 RRRr*	12/36 RRrr	3/36 Rrrr
3/6 rr	*3/36 RRrr*	12/36 Rrrr	3/36 rrrr

	Observed	Expected
RRrr×rrrr		
RRrr	84	86
Rrrr	101	107.5
rrrr	30	21.5

B^2^			
Male gametes	Female gametes		
	*1/6 RR*	4/6 Rr	1/6 rr
6/6 rr	*6/6 RRrr*	24/6 Rrrr	6/6 rrrr

	Observed	Expected
Rrrr×rrrr		
Rrrr	136	128.8
rrrr	25	32.2

Italic cells were zeroed for the tested hypothesis. (A) CT population: unviable gametes when homozygous for the *Ry*_*adg*_ resistant gene resulting in segregation (1N:5S:4D). (B) TL population: unviable gametes when homozygous for the *Ry*_*adg*_ resistant gene resulting in segregation (1N:4S)

^1^The Chi value is 3.8. The *p* value is 0.15. The result is not significant at *p* ≤ 0.05

^2^The Chi value is 2.012. The *p* value is 0.156. The result is not significant at *p* ≤ 0.05

To corroborate the HRM results, 34 genotypes of the CT population representing nulliplex, simplex, duplex and triplex were phenotyped by mechanical inoculation of PVY°. The LBr-43 genotype was included as control. Visual observation and DAS-ELISA showed the presence of PVY only in the LBr-43 genotype and not in the nulliplex genotypes of the CT population. Hence, the TXY.2 parent of the CT population could carry another PVY resistance gene distinct from *Ry*_*adg*_ at the M6 locus. To test this hypothesis, a new population (TL) was developed crossing TXY.2 (T) and the susceptible genotype LBr-43 (L) to test the presence of another PVY-resistant gene.

### HRM allele-dosage assay and PVY resistance test on the TL population

The M6 marker produced the expected amplicon in 66 out of 81 genotypes of the TL population by using the new M6 primers (M6F1 and M6R4). This segregation ratio corresponds to the expected frequency (1:5) for the progeny of a cross between a nulliplex and a duplex. The HRM allele-dosage assay was performed on 163 progenies including the simplex variety Costanera as control. Two clearly separated peaks corresponding to the resistant M6R and the three susceptible M6S1,2,3 alleles were identified (Fig. 4c). Of the 163 samples, 2 did not amplify, 25 grouped as nulliplex and 136 as simplex. No duplex genotypes were observed. The Chi square goodness of fit test did not support the hypothesis that the observed values correspond to the expected segregation ratio from a cross between a nulliplex and a duplex (1N:4S:1D). Hence, we retested the same hypothesis of unviable gamete when homozygous for the *Ry*_*adg*_ resistant gene resulting in a segregation (1N:4S). The Chi square goodness of fit test (*x*^2^ = 2.012; *p *= 0.15; *p* ≤ 0.05) failed to reject this alternative hypothesis (Table 4b).

Ten nulliplex, ten simplex and controls TXY.2 and LBr-43 were mechanically inoculated with PVY°. Three nulliplex were positive for PVY° for the DAS-ELISA test and showed symptoms of infection while LBr-43 was positive for PVY°, although did not show clear symptoms of infection. Seven of the nulliplex were negative for PVY° for the DAS-ELISA test and did not show symptoms of infection. Hence, this second progeny testing of nulliplex for *Ry*_*adg*_ displaying resistance to PVY indicates the presence of another resistance gene in the TXY.2 progenitor.

## Discussion

The PVY resistance phenotype was confirmed for all the material and coincided with previous assessments. As evidenced with the varieties Desiree and Perricholi, the phenotyping of extreme resistance must be assessed by both symptoms and DAS-ELISA. The marker analyses coincided also with our knowledge on virus resistance and pedigree of these varieties and breeding lines.

The marker RYSC3 developed originally from an accession of *S. tuberosum* L. group Andigena displayed the marker allele associated with resistance for all breeding materials derived from the Andigena XY progenitors (UNICA, Costanera, DXY.10, DXY.15, TXY.2, and TXY.11). However, the RYSC3 marker allele associated with resistance was also present in the clone A6 which is susceptible to PVY and used for PVY strain identification. It is unclear yet from pedigree information why such marker allele is present in the A6 clone. RYSC3 was absent in the PVY-resistant I-1039 cultivar unlike the other markers. This low association of RYSC3 marker with *Ry*_*adg*_ gene was also reported for two varieties from Uruguayan breeding material with extreme resistance assumed to be controlled by the *Ry*_*adg*_ gene based on pedigree information (Dalla Rizza et al. 2006). These findings indicate the existence of a lower genetic linkage between RYSC3 marker and the *Ry*_*adg*_ gene on the chromosome XI than the other markers.

The markers M5, M6, M17 and M45 displayed marker alleles associated with resistance not only in the parental material they were originally developed from, I-1039, but also in all of the breeding material derived from the Andigena XY progenitors. This result supports that the *Ry*_*adg*_ and *Ry*_*sto*_ genes map at the same locus, but whether these are distinct genes or allelic variants of the same gene is unknown. In addition, the differences in symptoms to distinguish extreme and hypersensitive resistance may not be so clear and the recently found *Ny*-*2* and *Ny*_*(o,n)sto*_ genes on chromosome XI may be the same as *Ry*_*sto*_ found by Brigneti et al. (1997).

In *S. stoloniferum*, the RYSC3 and M45 marker alleles are detected in the susceptible but not the resistant *sto* accession, whereas the M6 marker is present in the resistant but absent in the susceptible accession. Hence, these markers are not associated with *S. stoloniferum* resistance to PVY, which is likely to be determined by the *Ry*-*f*_*sto*_ gene on chromosome XII.

The demonstration that the *Ry*_*adg*_ gene on chromosome XI in our breeding lines originates from the Andigena group and not from the *S. stoloniferum* wild species was made by characterizing the DNA sequence at the M6 and M45 tightly linked markers and by screening a sample of the Andigena germplasm. Indeed, the identification of an identical resistant allele for the M6 locus (M6R) in Costanera, UNICA and I-1039, which is different from the other susceptible alleles including those from *S. stoloniferum*, supports the hypothesis of a common origin of the resistant allele M6R. This result coincides with the molecular characterization of I-1039 by SSR markers and the absence of the Tuberosum plastid marker, which placed this cultivar as a hybrid of Andigena × Tuberosum (Ghislain et al. 2009). Sequence analyses of the M6 and M45 alleles from Costanera, UNICA and I-1039 confirmed the high similarity between them suggesting again a common origin of these alleles. Secondly, we screened a randomly chosen sample of landraces of the Andigena group with the markers associated with *Ry*_*adg*_ gene on chromosome XI. One landrace out of 251 screened was positive for all three markers and tested resistant to PVY which reveal that this *Ry*_*adg*_ gene is present in the Andigena group. Hence, we conclude here that the *Ry*_*sto*_ gene of I-1039 on chromosome XI maps at the same locus as the *Ry*_*adg*_ gene, but whether these are identical or close by genes remain to be demonstrated by sequencing larger region around this locus.

To develop a quick and efficient marker-assisted diagnostic for the presence/absence of the *Ry*_*adg*_ gene, we have developed new primers and amplification conditions to amplify more reliably M6 and M45 markers. It has been calculated that 60% of the total time required from leaf collection to PCR reaction is used for DNA extraction (Dilworth and Frey 2000). Thus, simple, rapid, robust and inexpensive DNA isolation method is needed for high-throughput MAS (von Post et al. 2003; Karakousis and Langridge 2003). Unlike other plant DNA extraction protocols, the method implemented in this study does not include the use of liquid nitrogen or freeze-drying for initial grinding of the tissue. Furthermore, it does not require hazardous chloroform precipitation or sophisticated automated equipment used by large breeding companies. However, this protocol is not suitable for isolating large quantities of DNA which may limit its use if multiple marker assays must be performed sequentially. The cost of DNA extraction, PCR amplification and markers detection are also considered as restricting factors for application of MAS in breeding programs (Xu and Crouch 2008). In addition to technical and safety advantages, the reported protocol for DNA extraction is a cost-effective method. In our laboratory, the cost per sample following routine protocols is an order magnitude higher and takes 6 h to process 48 samples. Optimization of multiplex PCR requires strategic planning and multiple attempts to avoid results that can lead to false negatives or positives. Our results demonstrated that the protocol for the multiplex PCR developed in this study permitted considerable savings of time and effort without compromising accuracy. This protocol was used for identifying recombinant genotypes in the CL segregating population developed to accomplish the fine mapping of the *Ry*_*adg*_ locus (Jara Vidalon 2010). However, the specificity of this multiplex *Ry*_*adg*_ markers should be characterized using cultivars with other PVY resistance genes from *S stoloniferum* such as those mentioned in Van Eck et al. (2017), Tomczyńska et al. (2014), Mori et al. (2011), and Szajko et al. (2008, 2014).

The rapid introgression of the *Ry*_*adg*_ gene into promising breeding lines would be greatly improved by increasing the frequency of this allele in breeding population by using progenitors with multiple copies of this allele. We developed here an allele-dosage assay for the tightly linked M6 marker based on high-resolution melting technology using unlabelled probes instead of fluorogenic 5′ nuclease (TaqMan) for two reasons: (1) it is more cost-effective because it does not require expensive labeled oligonucleotide probes; and (2) it can detect multiple haplotypes in a single assay unlike the HRM-TaqMan 5′ assay which only detects a single allele relative to any another allele. Indeed, HRM-probe genotyping was used to select parents with multiple copies of a desirable marker (De Koeyer et al. 2010). Probe design for a perfect complementation with the resistant allele allowed differentiating the haplotypes present in the region of the probe. The HRM allele-dosage assay for the *Ry*_*adg*_ gene developed in this study allowed easy differentiation of resistant samples from the susceptible ones and clear identification of nulliplex, simplex duplex and triplex genotypes without having to do the tedious phenotypic assay on segregating progenies.

Surprisingly, all multiplex PVY-resistant progenitors tested appeared to have a lower *Ry*_*adg*_ allele dosage than what had been observed by progeny analyses. The latter may have overestimated the allele-dosage number likely due to unsuccessful PVY infection or additional PVY resistance genes. Indeed, the duplex status of the triplex progenitor TXY.2 was confirmed by the allele-dosage assay for both CT and TL populations, and by the segregation ratio of M6 marker in the TL population. This indicates that the allele-dosage level previously assigned to the *Ry*_*adg*_ gene was incorrect for all duplex and triplex samples of the panel. The absence of PVY disease symptoms and positive results for PVY° with DAS-ELISA in none of the eight nulliplex of the CL population and in seven out of ten nulliplex genotypes from the TL population suggest that another resistant gene to PVY might be present in TXY.2. Solomon-Blackburn and Mackay (1993) showed that PVY susceptible varieties can be symptomless. We cannot rule out some failure in the infection process. This would explain the presence of asymptomatic leaves on some nulliplex genotypes of the CT and TL populations. The quantity of PVY present in these genotypes could not be detected with DAS-ELISA as suggested by Depta et al. (2014). These observations reinforce the importance of having molecular methods for PVY resistance diagnostic to complement the phenotypic detection methods.

In conclusion, the rapid protocol for DNA extraction and multiplex PCR constitute a medium-throughput system for assessing the presence of RYSC3, M6 and M45 markers in potato allowing unequivocal identification of potato material with the resistant allele of the *Ry*_*adg*_ gene. The HRM allele-dosage assay proved to be robust for allele-dosage determination of *Ry*_*adg*_-linked marker M6. Both assays have proven to be promising tools to facilitate the introgression of the *Ry*_*adg*_ gene and would be a good alternative for breeding programs with limited budget. However, as indicated by Cernák et al. (2008), the genetic background is critical and must be take into consideration for the applicability of this molecular marker system.

### Author contribution statement

MRH and MG directed the experiments, LJV and JM conducted the plex assay and population development, IB conducted the PVY resistance assay, CR and FG did the marker allele sequence analyses, LJV developed the multiplex marker assay and population screening and MRH, LJV and MG wrote the manuscript.
